# InforMing the PAthway of COPD Treatment (IMPACT) trial: fibrinogen levels predict risk of moderate or severe exacerbations

**DOI:** 10.1186/s12931-021-01706-y

**Published:** 2021-04-28

**Authors:** Dave Singh, Gerard J. Criner, Mark T. Dransfield, David M. G. Halpin, MeiLan K. Han, Peter Lange, Sally Lettis, David A. Lipson, David Mannino, Neil Martin, Fernando J. Martinez, Bruce E. Miller, Robert Wise, Chang-Qing Zhu, David Lomas

**Affiliations:** 1 Centre for Respiratory Medicine and Allergy, Institute of Inflammation and Repair, Manchester Academic Health Science Centre, The University of Manchester, Manchester University NHS Foundation Trust, Manchester, UK; 2 Pulmonary and Critical Care Medicine, Lewis Katz School of Medicine at Temple University, Philadelphia, PA USA; 3 Division of Pulmonary, Allergy, and Critical Care Medicine, Lung Health Center, University of Alabama at Birmingham, Birmingham, AL USA; 4 University of Exeter Medical School, College of Medicine and Health, University of Exeter, Exeter, UK; 5 University of Michigan, Pulmonary & Critical Care, Ann Arbor, MI USA; 6 Department of Public Health, University of Copenhagen, Copenhagen, Denmark; 7 Biostatistics, GlaxoSmithKline, Stockley Park West, Uxbridge, Middlesex, UK; 8 Clinical Sciences, GlaxoSmithKline, Collegeville, PA USA; 9 Pulmonary, Allergy and Critical Care Division, Department of Medicine, Perelman School of Medicine, University of Pennsylvania, Philadelphia, PA USA; 10 University of Kentucky College of Public Health, Lexington, KY USA; 11 Global Medical Affairs, GlaxoSmithKline, Brentford, Middlesex, UK; 12 Institute for Lung Health, University of Leicester, Leicester, UK; 13 Weill Cornell Medicine, New York, NY USA; 14 Division of Pulmonary and Critical Care Medicine, Johns Hopkins University School of Medicine, Baltimore, MD USA; 15 Division of Medicine, UCL Respiratory, Rayne Building, University College London, London, WC1E 6BN UK

**Keywords:** Fibrinogen, COPD exacerbations, Pharmacotherapy, COPD

## Abstract

**Background:**

Fibrinogen is the first qualified prognostic/predictive biomarker for exacerbations in patients with chronic obstructive pulmonary disease (COPD). The IMPACT trial investigated fluticasone furoate/umeclidinium/vilanterol (FF/UMEC/VI) triple therapy versus FF/VI and UMEC/VI in patients with symptomatic COPD at risk of exacerbations. This analysis used IMPACT trial data to examine the relationship between fibrinogen levels and exacerbation outcomes in patients with COPD.

**Methods:**

8094 patients with a fibrinogen assessment at Week 16 were included, baseline fibrinogen data were not measured. Post hoc analyses were performed by fibrinogen quartiles and by 3.5 g/L threshold. Endpoints included on-treatment exacerbations and adverse events of special interest (AESIs).

**Results:**

Rates of moderate, moderate/severe, and severe exacerbations were higher in the highest versus lowest fibrinogen quartile (0.75, 0.92 and 0.15 vs 0.67, 0.79 and 0.10, respectively). The rate ratios (95% confidence interval [CI]) for exacerbations in patients with fibrinogen levels ≥ 3.5 g/L versus those with fibrinogen levels < 3.5 g/L were 1.03 (0.95, 1.11) for moderate exacerbations, 1.08 (1.00, 1.15) for moderate/severe exacerbations, and 1.30 (1.10, 1.54) for severe exacerbations. There was an increased risk of moderate/severe exacerbation (hazard ratio [95% CI]: highest vs lowest quartile 1.16 [1.04, 1.228]; ≥ 3.5 g/L vs < 3.5 g/L: 1.09 [1.00, 1.16]) and severe exacerbation (1.35 [1.09, 1.69]; 1.27 [1.08, 1.47], respectively) with increasing fibrinogen level. Cardiovascular AESIs were highest in patients in the highest fibrinogen quartile.

**Conclusions:**

Rate and risk of exacerbations was higher in patients with higher fibrinogen levels. This supports the validity of fibrinogen as a predictive biomarker for COPD exacerbations, and highlights the potential use of fibrinogen as an enrichment strategy in trials examining exacerbation outcomes.

*Trial registration*: NCT02164513

**Supplementary Information:**

The online version contains supplementary material available at 10.1186/s12931-021-01706-y.

## Background

Chronic obstructive pulmonary disease (COPD), a common disease characterized by persistent respiratory symptoms and airflow limitation, is associated with a substantial economic and social burden and was the third leading cause of death in 2017 worldwide [1, 2]. The InforMing the PAthway of COPD Treatment (IMPACT) study compared once-daily single-inhaler triple therapy with fluticasone furoate, umeclidinium and vilanterol (FF/UMEC/VI) with the dual therapies FF/VI and UMEC/VI in patients with symptomatic COPD at risk of exacerbations. This study demonstrated that FF/UMEC/VI resulted in a significantly lower annual rate of moderate/severe COPD exacerbations and greater improvements in both lung function and health-related quality of life compared with either dual therapy [3].

Forced expiratory volume in 1 s (FEV_1_) is a widely used lung function marker of COPD disease severity, but correlates poorly with symptoms and does not reflect levels of disease activity [2, 4–6]. Patients with more active COPD disease are more likely to experience faster disease progression; for example, patients with more exacerbations experience a faster decline in lung function [7, 8]. Therefore, alternative and more sensitive biomarkers are needed that are reflective of disease activity, and may predict future risk of events such as exacerbations. Fibrinogen is an acute phase soluble plasma glycoprotein that is primarily synthesized in the liver and is converted into fibrin by thrombin during blood coagulation [4]. Plasma fibrinogen was qualified by the US Food and Drug Administration (FDA) in 2015 as the first prognostic or enrichment biomarker for exacerbations and/or all-cause mortality (ACM) in patients with COPD [9, 10]. This validation was based on an analysis of an integrated study database, which demonstrated that high baseline fibrinogen levels in individuals with COPD were associated with an increased risk of exacerbations requiring hospitalization within 12 months and an increased risk of death within 36 months [11, 12]. Previous research on a subset of the ECLIPSE cohort indicated that fibrinogen was the most stable of 34 biomarkers assessed over a period of 3 months, a critical feature for clinically useful biomarkers [4, 13]. Additionally, fibrinogen is routinely measured in clinical practice, and therefore appears to be a practical blood biomarker for systemic inflammation in COPD [4]. A fibrinogen level of 3.5 g/L has been proposed as a threshold for identifying patients at increased risk of exacerbations requiring hospitalization and ACM [11].

This analysis used data collected in the IMPACT trial to provide further information regarding the association of fibrinogen levels with exacerbations and ACM in patients with COPD.

## Methods

### Study design and population

The IMPACT trial (study CTT116855, NCT02164513) was a Phase III, randomized, double-blind, parallel-group, multicenter study conducted in 37 countries between June 2014 and July 2017. The study design has been described in detail previously [3]. Briefly, the total study duration was approximately 55 weeks, consisting of a 2-week run-in period, 52-week treatment period, and a 1-week safety follow-up period. Study patients were randomized (2:2:1) to receive triple therapy with FF/UMEC/VI 100/62.5/25 µg, or dual therapy with FF/VI 100/25 µg or UMEC/VI 62.5/25 µg administered once daily via the Ellipta dry powder inhaler. Patients continued use of their existing COPD medications during the run-in period and were provided with albuterol for use on an as-needed basis (rescue medication) throughout the study. All patients provided written informed consent. The study was conducted in accordance with Good Clinical Practice guidelines and the provisions of the Declaration of Helsinki and received approval from local institutional review boards or independent ethics committees.

Inclusion/exclusion criteria have been described previously [3, 14]. Eligible patients were ≥ 40 years of age, with symptomatic COPD (COPD Assessment Test score ≥ 10) and either a post-bronchodilator FEV_1_ < 50% of the predicted normal value and a history of ≥ 1 moderate or severe exacerbation in the previous year, or a post-bronchodilator FEV_1_ of > 50 to < 80% of the predicted normal value and ≥ 2 moderate exacerbations or ≥ 1 severe exacerbation in the previous year.

The intent-to-treat (ITT) population comprised all randomized patients, excluding those randomized in error [14]. The fibrinogen population is a subset of the ITT population that included patients with a fibrinogen assessment at Week 16, fibrinogen data were not collected prior to Week 16. Previous work has shown that treatment with oral steroids or inhaled corticosteroid/long-acting β_2_-agonist (ICS/LABA) did not have an effect on fibrinogen levels [15, 16].

Annual rate of COPD exacerbations and ACM were analyzed post hoc in the fibrinogen population according to a fibrinogen threshold: ≥ or < 3.5 g/L and by fibrinogen quartiles (Quartile 1: < 2.780 g/L; Quartile 2: ≥ 2.780 to < 3.280 g/L; Quartile 3: ≥ 3.280 to < 3.830 g/L; Quartile 4: ≥ 3.830 g/L). Analysis of safety was carried out using fibrinogen quartiles.

### Study endpoints

Study endpoints have been described previously, with the primary endpoint from the IMPACT trial being the annual rate of moderate/severe exacerbations during treatment [3, 14]. This analysis assessed the following endpoints from Week 16: on-treatment exacerbations (moderate, moderate/severe, severe), on- and off-treatment ACM and on-treatment adverse events of special interest (AESIs). A moderate exacerbation was defined as an exacerbation leading to treatment with antibiotics or systemic corticosteroids. A severe exacerbation was one resulting in hospitalization or death. On-/off-treatment deaths were defined as those that occurred after Week 16 and study treatment stop date and projected Week 52 date (based on treatment start date) plus 7 days (inclusive).

This analysis evaluated the association between fibrinogen levels at Week 16 and exacerbations, mortality and AESIs, in particular cardiovascular (CV) events and pneumonia. To confirm that Week 16 fibrinogen could be used as a baseline value for exacerbation analyses, we assessed the magnitude of the difference in Week 16 fibrinogen between the treatment groups to check whether fibrinogen levels were affected by treatment. Furthermore, to assess whether the Week 16 fibrinogen population was generalizable to all patients we evaluated exacerbation history between the patients who withdrew at Week 16 versus those who remained in the study.

### Statistical analyses

These post hoc analyses were performed in two stages. First, baseline characteristics were summarized in the fibrinogen population using screening and baseline visit data. Analysis of variance (ANOVA) was used to assess the magnitude of differences in fibrinogen levels at Week 16 between treatment groups. The Scheffe multiple comparison procedure was used to compare the difference between treatment groups. The second stage includes all other analyses. Fibrinogen data collected at Week 16 was considered as baseline in this post hoc analysis. All analyses were performed using endpoint data from Week 16 onwards. For example, only exacerbations that occurred after Week 16 were considered as an event in the analysis of COPD exacerbations annual rate and the analysis of time-to-first COPD exacerbation.

The rate of on-treatment COPD exacerbations (moderate/severe, moderate, severe) was analyzed using a generalized linear model assuming a negative binomial distribution. Rate of on-treatment COPD exacerbation analyses were performed by comparing fibrinogen subgroups (fibrinogen threshold [3.5 g/L] and fibrinogen quartiles) with combined treatment groups and by comparing treatment groups for each fibrinogen subgroup (fibrinogen threshold [3.5 g/L] and fibrinogen quartiles). The time-to-first on-treatment COPD exacerbation (moderate, moderate/severe, severe) was analyzed using a Cox proportional hazards model by comparing fibrinogen subgroups with combined treatment groups. The relationship between exacerbation rate and fibrinogen was also explored by using fractional polynomials modelling, in which fibrinogen was included as a continuous variable in the model. The analyses of exacerbation rates comparing fibrinogen subgroups included covariates of treatment group, gender, exacerbation history in the past 12 months (≤ 1, ≥ 2 moderate/severe), smoking status (at screening), geographical region and post-bronchodilator percent predicted forced expiratory volume in 1 s (FEV1; at screening) and fibrinogen quartile group or 3.5 g/L threshold as applicable. The fibrinogen subgroup analyses of exacerbation rates comparing treatment groups included covariates of treatment group, gender, exacerbation history in the past 12 months (≤ 1, ≥ 2 moderate/severe), smoking status (at screening), geographical region and post-bronchodilator percent predicted FEV1 (at screening). Patients were only included in the model if they had at least 1 day at risk of an on-treatment exacerbation from Week 16. The time at risk excluded any days after Week 16 when a patient was experiencing an exacerbation that started on or prior to Week 16. Analysis by continuous fibrinogen level was performed using fractional polynomials and included covariates of treatment group, gender, exacerbation history (≤ 1, ≥ 2 moderate/severe), smoking status (at screening), geographical region, post-bronchodilator percent predicted FEV_1_ (at screening), FP1, FP2, interaction terms for FP1 by treatment, FP2 by treatment, and with logarithm of time on-treatment post Week 16 as an offset variable. FP1 and FP2 represent continuous transformations of fibrinogen at Week 16. Subgroup models by fibrinogen quartile at Week 16 with the same covariates but excluding FP terms are overlaid on the plots at the subgroup median fibrinogen value. Time to on- and off- treatment ACM was analyzed by calculation of Kaplan–Meier probability estimates for each fibrinogen subgroup. Adverse events (AEs), serious adverse events (SAEs) and AESIs were summarized using descriptive statistics.

## Results

Of the 10,355 patients enrolled in the IMPACT study, 8094 had Week 16 fibrinogen data available and were included in this analysis (Table 1). Demographics and baseline characteristics were generally similar across Week 16 fibrinogen quartile or 3.5 g/L threshold subgroups and consistent with the ITT population (Table 1). Baseline characteristics by fibrinogen subgroup and treatment arms are presented in Table 1 in Additional File 1 (quartiles) and Table 2 in Additional File 1 (3.5 g/L threshold). The median (interquartile range [IQR]) overall (all treatment combined) fibrinogen level at Week 16 was 3.280 (2.780–3.830) g/L.

**Table 1:** Baseline characteristics and demographics by fibrinogen subgroup at Week 16

Characteristic	Fibrinogen population							ITT population
	Quartile 1 n = 2017 < 2.780^a^	Quartile 2 n = 2025 ≥ 2.780 to < 3.280^a^	Quartile 3 n = 2002 ≥ 3.280 to < 3.830^a^	Quartile 4 n = 2050 ≥ 3.830^a^	< 3.5 g/L n = 5045	≥ 3.5 g/L n = 3049	Total N = 8094	Total N = 10,355
Age, years, mean (SD)	65.2 (8.4)	65.0 (8.3)	65.2 (8.2)	65.5 (8.1)	65.2 (8.3)	65.3 (8.1)	65.2 (8.2)	65.3 (8.27)
Male, n (%)	1516 (75)	1345 (66)	1269 (63)	1356 (66)	3503 (69)	1983 (65)	5846 (68)	6870 (66)
BMI, kg/m^2^, mean (SD)	25.4 (5.3)	26.3 (5.8)	27.2 (6.2)	27.6 (6.6)	26.1 (5.6)	27.6 (6.6)	26.6 (6.1)	26.6 (6.1)
Smoking status, n (%)								
Current	554 (27)	676 (33)	787 (39)	785 (38)	1614 (32)	1188 (39)	2802 (35)	3587 (35)
Former	1463 (73)	1349 (67)	1215 (61)	1265 (62)	3431 (68)	1861 (61)	5292 (65)	6768 (65)
Lung function (post-bronchodilator)								
FEV_1_ (L), mean (SD)	1.348 (0.4929)	1.315 (0.4923)	1.275 (0.4833)	1.241 (0.4652)	1.320 (0.4914)	1.251 (0.4716)	1.294 (0.4851)	1.272 (0.4860)
FEV_1_% predicted, mean (SD)	48.0 (14.9)	46.8 (14.6)	45.7 (14.8)	44.4 (14.3)	47.1 (14.8)	44.7 (14.5)	46.2 (14.7)	45.5 (14.8)
FEV_1_/FVC ratio, mean (SD)	0.472 (0.1185)	0.475 (0.1187)	0.474 (0.1193)	0.473 (0.1192)	0.473 (0.1182)	0.475 (0.1201)	0.474 (0.1189)	0.470 (0.1196)
COPD exacerbations in previous year, n (%)								
Moderate								
0	367 (18)	370 (18)	354 (18)	397 (19)	916 (18)	572 (19)	1488 (18)	1936 (19)
1	636 (32)	701 (35)	705 (35)	696 (34)	1681 (33)	1057 (35)	2738 (34)	3542 (34)
≥ 2	1014 (50)	954 (47)	943 (47)	957 (47)	2448 (49)	1420 (47)	3868 (48)	4877 (47)
Moderate/severe								
0	3 (< 1)	1 (< 1)	1 (< 1)	1 (< 1)	5 (< 1)	1 (< 1)	6 (< 1)	9 (< 1)
1	883 (44)	909 (45)	912 (46)	925 (45)	2247 (45)	1382 (45)	3629 (45)	4691 (45)
≥ 2	1131 (56)	1115 (55)	1089 (54)	1124 (55)	2793 (55)	1666 (55)	4459 (55)	5655 (55)
Severe								
0	1532 (76)	1507 (74)	1500 (75)	1501 (73)	3796 (75)	2244 (74)	6040 (75)	7684 (74)
1	423 (21)	451 (22)	438 (22)	467 (23)	1088 (22)	691 (23)	1779 (22)	2300 (22)
≥ 2	62 (3)	67 (3)	64 (3)	82 (4)	161 (3)	114 (4)	275 (3)	371 (4)
Current medical conditions, n (%)								
Angina pectoris	50 (2)	75 (4)	73 (4)	80 (4)	161 (3)	117 (4)	278 (3)	342 (3)
Myocardial infarction	0	0	0	0	0	0	0	1 (< 1)
Cardiovascular risk factors^b^, n (%)								
Angina pectoris	111 (6)	160 (8)	147 (7)	162 (8)	346 (7)	234 (8)	580 (7)	737 (7)
Previous myocardial infarction	112 (6)	117 (6)	138 (7)	166 (8)	308 (6)	225 (7)	533 (7)	681 (7)

Quartile 1, fibrinogen value < 25th percentile; Quartile 2, fibrinogen value > 25th percentile and < median; Quartile 3, fibrinogen value ≥ median and < 75th percentile; Quartile 4, fibrinogen value ≥ 75th percentile

*BMI* body mass index, *SD* standard deviation

^a^Fibrinogen level descriptor (g/L)

^b^Risk factors includes past and current events

### Week 16 and baseline comparison

Exacerbation history in the 12 months prior to screening was similar between patients who withdrew before Week 16 and those who remained in the study (Table 3 in Additional File 1). Table 2 summarizes exacerbations experienced within the first 16 weeks of the trial: 22–30% of patients who continued in the trial, and had Week 16 fibrinogen data, experienced an exacerbation within the first 16 weeks; 46% of patients who withdrew from the trial on or prior to Week 16, and therefore did not have fibrinogen data, experienced an exacerbation within this time period. Differences between Week 16 fibrinogen levels in patients who received FF/UMEC/VI versus FF/VI (1%), FF/UMEC/VI versus UMEC/VI (2%), and UMEC/VI versus FF/VI (− 1%) were below the pre-specified ≤ 15% indicating no evidence of a potential treatment effect on Week 16 fibrinogen (Table 4 in Additional File 1). Based on these results Week 16 Fibrinogen was considered appropriate to use as baseline measurement.

**Table 2:** Exacerbation status up to Week 16 by fibrinogen quartile and withdrawal status

	Week 16 fibrinogen data				No Week 16 fibrinogen data		
Exacerbation	Quartile 1 N = 2017	Quartile 2 N = 2025	Quartile 3 N = 2002	Quartile 4 N = 2050	Withdrew^a^ N = 1195	Other^b^ N = 1066	Total N = 10,355
Yes, n (%)	452 (22)	476 (24)	509 (25)	609 (30)	547 (46)	271 (25)	2864 (28)
No, n (%)	1565 (78)	1549 (76)	1493 (75)	1441 (70)	648 (54)	795 (75)	7491 (72)

Quartile 1, fibrinogen value < 25th percentile; Quartile 2, fibrinogen value > 25th percentile and < median; Quartile 3, fibrinogen value ≥ median and < 75th percentile; Quartile 4, fibrinogen value ≥ 75th percentile

^a^Patients who discontinued study treatment/withdrew from the study on or prior to Week 16 and did not have a fibrinogen result at Week 16;

^b^Patients who did not discontinue study treatment/withdraw from the study on or prior to Week 16 but had a missing fibrinogen result at Week 16

### Efficacy

#### On-treatment exacerbation rates

**Fig. 1:**
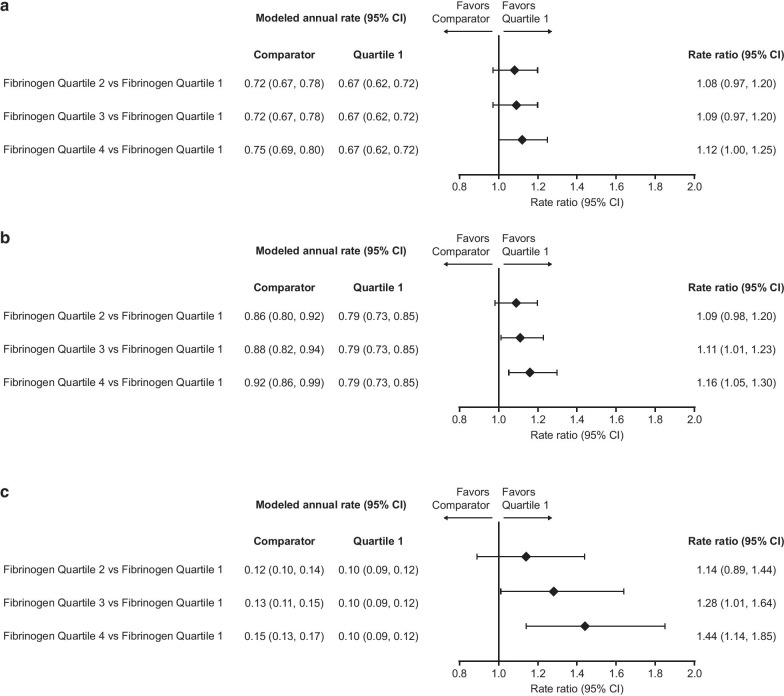
Rate of on-treatment exacerbations by quartiles. **a** Moderate; **b** moderate/severe; **c** severe. Rate of exacerbations from Week 16 by Week 16 fibrinogen quartiles. Quartile 1, fibrinogen value < 25th percentile; Quartile 2, fibrinogen value > 25th percentile and < median; Quartile 3, fibrinogen value ≥ median and < 75th percentile; Quartile 4, fibrinogen value ≥ 75th percentile; interaction of treatment with fibrinogen quartile p-values for **a**–**c** are 0.494, 0.515 and 0.916 respectively. *CI* confidence interval

**Fig. 2:**
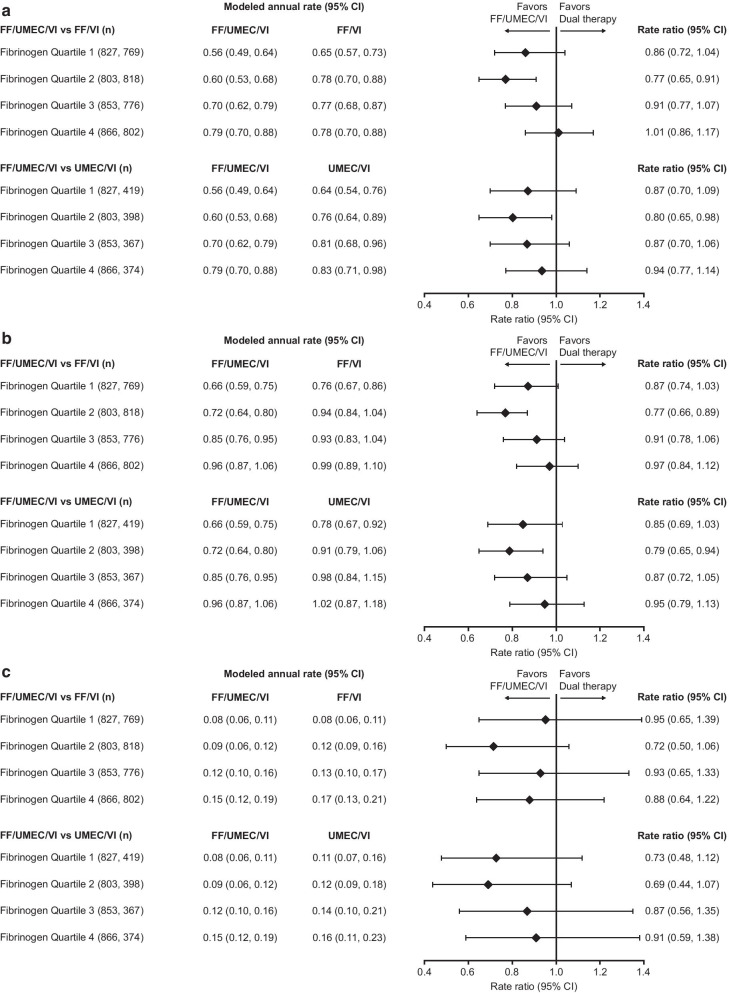
Rate ratio for on-treatment exacerbations by quartile. **a** Moderate; **b** moderate/severe; **c** severe. Rate ratio (FF/UMEC/VI vs dual therapies) for on-treatment exacerbations by Week 16 fibrinogen quartile. Quartile 1, fibrinogen value < 25th percentile; Quartile 2, fibrinogen value > 25th percentile and < median; Quartile 3, fibrinogen value ≥ median and < 75th percentile; Quartile 4, fibrinogen value ≥ 75th percentile. *CI* confidence interval; *FF* fluticasone furoate; *n* number of patients in the analysis in each subgroup; *UMEC* umeclidinium; *VI* vilanterol

The rates of moderate, moderate/severe, and severe exacerbations increased with increasing fibrinogen quartiles (Fig. 1). Significant differences (indicated by 95% confidence interval [CI] that did not cross 1) were observed between the rates of moderate/severe and severe exacerbations for Quartile 4 versus Quartile 1, with rate ratios of 1.16 and 1.44, respectively (Fig. 1). Similarly, a significant difference was also observed between Quartile 3 and Quartile 1, with rate ratios of 1.11 and 1.28, for moderate/severe and severe exacerbations, respectively (Fig. 1). For moderate exacerbations, the difference was significant only for Quartile 4 versus Quartile 1 (rate ratio 1.12) (Fig. 1). When analyzed by treatment arm in each quartile subgroup, the rate ratio point estimates for moderate, moderate/severe, and severe exacerbations were generally in favor of FF/UMEC/VI over each dual therapy (Fig. 2).

**Fig. 3:**
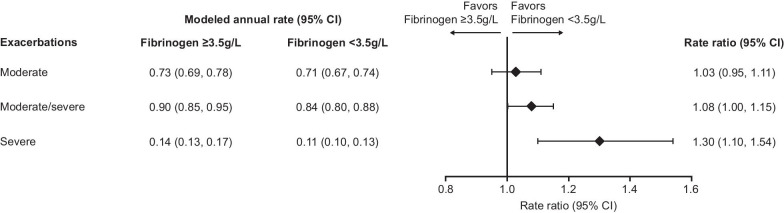
Rate of on-treatment exacerbations by 3.5 g/L threshold. Rate of on-treatment exacerbations from Week 16 by Week 16 fibrinogen 3.5 g/L threshold. *CI* confidence interval

**Fig. 4:**
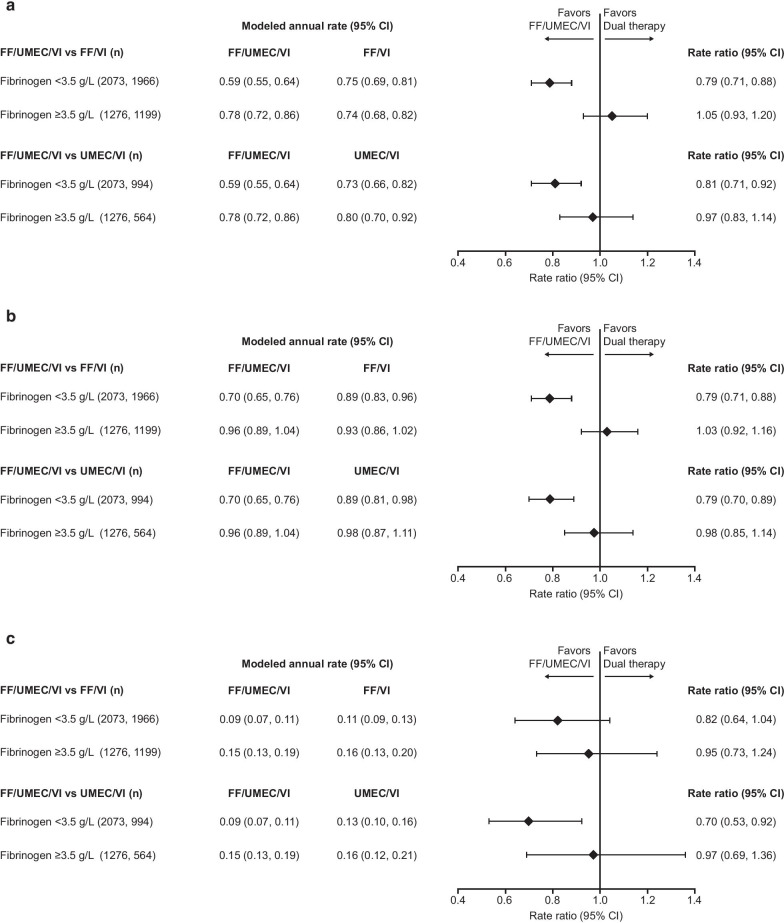
Rate ratio for on-treatment exacerbations by 3.5 g/L threshold. **a** Moderate; **b** moderate/severe; **c** severe. Rate ratio (FF/UMEC/VI vs dual therapies) for on-treatment exacerbations by Week 16 fibrinogen 3.5 g/L threshold. *CI* confidence interval; *FF* fluticasone furoate; *n* number of patients in the analysis in each subgroup; *UMEC* umeclidinium; *VI* vilanterol

Exacerbation rates were also higher in patients with fibrinogen levels ≥ 3.5 g/L versus < 3.5 g/L, with rate ratios of 1.08 for moderate/severe exacerbations and 1.30 for severe exacerbations (Fig. 3). Analysis by treatment group in each fibrinogen threshold subgroup showed that FF/UMEC/VI reduced the rate of moderate, moderate/severe, and severe exacerbation versus both FF/VI and UMEC/VI in the low fibrinogen subgroup (< 3.5 g/L); no significant between-treatment differences were seen in patients with fibrinogen ≥ 3.5 g/L (Fig. 4).

Analysis of the rate of on-treatment moderate, moderate/severe, and severe exacerbations by continuous fibrinogen levels showed a general trend for an increased rate of exacerbations with increasing fibrinogen levels in all treatment arms and for all exacerbation types (Fig. 1 in Additional File 1).

#### Time-to-first on-treatment exacerbation

**Fig. 5:**
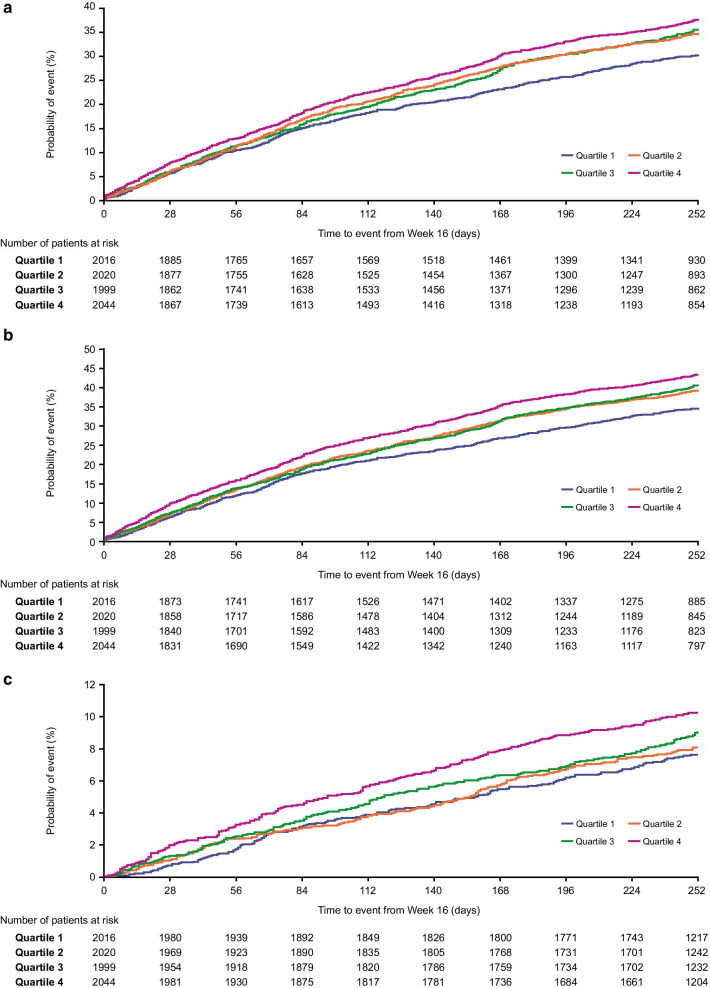
Time-to-first on-treatment exacerbation by quartile. **a** Moderate; **b** moderate/severe; **c** severe. Time-to-first on-treatment exacerbation from Week 16 by Week 16 fibrinogen quartile. Interaction of treatment with fibrinogen quartile p-values for **a**–**c** are 0.695, 0.599 and 0.783 respectively: Quartile 1: < 2.780 g/L; Quartile 2: ≥ 2.780 g/L; Quartile 3: < 3.830 g/L; Quartile 4: ≥ 3.830 g/L

Kaplan–Meier plots of time-to-first exacerbation showed a general trend for increased risk of an on-treatment moderate, moderate/severe, or severe exacerbation with increasing fibrinogen levels (Fig. 5). A significantly higher risk of moderate/severe (hazard ratio [HR] [95% confidence interval (CI)]: 1.16 [1.04, 1.28]) and severe (HR: 1.35 [1.09, 1.69]) exacerbations was seen between patients in fibrinogen Quartile 4 compared with Quartile 1.

Analysis of time-to-first exacerbations using the 3.5 g/L fibrinogen threshold demonstrated a significantly higher risk of on-treatment moderate/severe (HR [95% CI]: 1.09 [1.00, 1.16]) and severe (HR: 1.27 [1.08, 1.47]) exacerbations in patients with fibrinogen levels ≥ 3.5 g/L compared with those with levels < 3.5 g/L (Fig. 2 in Additional File 1).

#### All-cause mortality

**Fig. 6:**
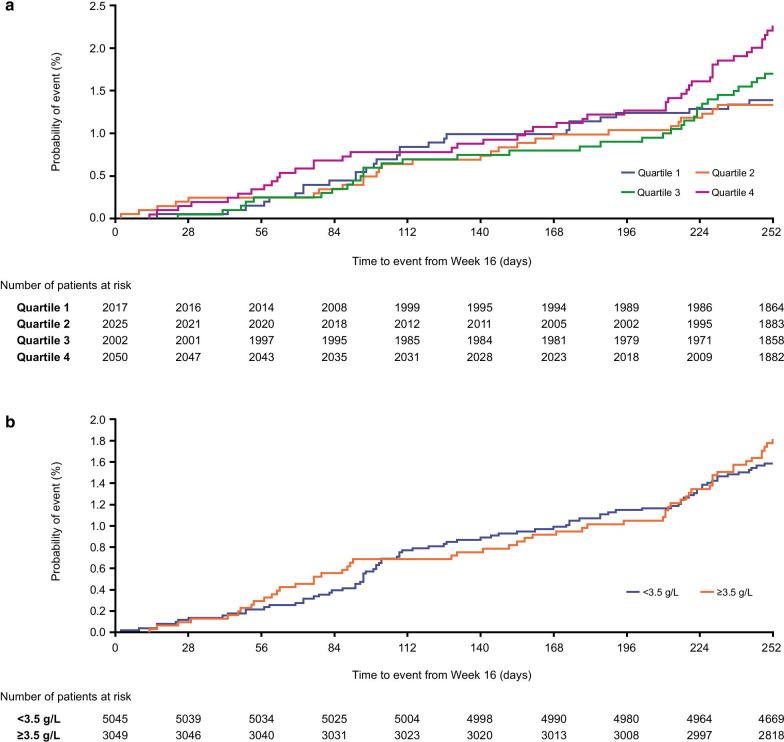
All-cause mortality on and off-treatment by **a** fibrinogen quartile; **b** fibrinogen 3.5 g/L threshold. Quartile 1: < 2.780 g/L; Quartile 2: ≥ 2.780 g/L; Quartile 3: < 3.830 g/L; Quartile 4: ≥ 3.830 g/L

Analysis of ACM showed a similar risk of on-/off-treatment mortality in the highest fibrinogen quartile group compared with the lower three quartiles (Fig. 6a). Similarly, analysis of ACM using the fibrinogen 3.5 g/L threshold showed a similar risk of on-/off-treatment mortality for fibrinogen levels below and above this threshold (Fig. 6b).

### Safety

Safety data from the IMPACT trial has been previously published [3]. In this current analysis, incidence of AESIs were similar across all fibrinogen quartiles. The incidence of pneumonia was similar (5%) in Quartiles 1, 2 and 3, but slightly higher (6%) in Quartile 4. For CV events, the overall incidence was similar in each quartile, but Quartile 4 had a slightly higher event rate (162.7) compared with Quartiles 1 (139.2), 2 (136.0) and 3 (129.4) (Table 5 in Additional File 1).

## Discussion

This post hoc analysis of the IMPACT trial was undertaken to investigate whether plasma fibrinogen could be used to stratify a high-risk COPD population and identify those most likely to have future events. Our findings indicate an increased rate and risk of exacerbation in patients with higher fibrinogen levels, which is seen regardless of whether fibrinogen is analyzed by quartiles or by a 3.5 g/L threshold. In this analysis, higher (≥ 3.5 g/L) versus lower (< 3.5 g/L) fibrinogen levels were associated with an 8% increase in exacerbations and a 30% increase in severe exacerbations (those leading to hospitalisation or death).

The results from this study support the rationale for using fibrinogen levels as a predictive biomarker for clinical trial recruitment as it provides an indicator of patients at an increased risk of COPD exacerbations [11]. Patients in the highest fibrinogen quartile had a 16% increase in the rate of moderate/severe exacerbations compared with the lowest quartile. This could be beneficial as an enrichment strategy in clinical trials looking at exacerbation outcomes, as more exacerbation outputs could be gained from fewer patients, and fewer patients would be required to demonstrate treatment effects. However, it is worth noting that by selecting patients in the highest fibrinogen quartile during clinical trial screening, 75% of the patient population would be excluded from participating. Importantly, findings from this study support the utility of fibrinogen as a predictive marker for exacerbations in clinical practice; aiding the identification of a subgroup of patients with COPD that are at a higher risk of experiencing exacerbations and would benefit from appropriate treatment to reduce future risk [4]. A general trend for increased risk of on-treatment moderate, moderate/severe, and severe exacerbations was observed with increasing fibrinogen levels for each exacerbation type, with a more pronounced change seen for severe exacerbations (i.e. those resulting in hospitalization or death), than moderate exacerbations. These results are in agreement with the studies by Mannino et al. [11] and Kim et al. [12], which reported that higher fibrinogen levels were associated with an increased risk of exacerbations in patients with COPD, and the study by Celli et al. [17], which demonstrated an increased risk of hospitalized exacerbations at higher fibrinogen levels, although no significant association was seen for moderate/severe exacerbations. The analysis of exacerbations by continuous fibrinogen levels provides further support for increased exacerbation rates in patients with higher fibrinogen levels.

The ACM data showed a similar risk of on-/off-treatment mortality when the highest fibrinogen quartile group was compared with the lower three quartiles and when the < 3.5 g/L and ≥ 3.5 g/L fibrinogen subgroups were compared. This differs from results shown in other studies that found that higher levels of plasma fibrinogen were associated with an increased risk of death [11, 17]. These differences could potentially be due to the fact that the Week 16 fibrinogen population may not represent all patients; furthermore, patients who died prior to Week 16, and therefore did not have fibrinogen data available, were not included in this analysis. Finally, it should be noted that this analysis considers a short duration of follow-up (from Week 16 to Week 52), and that there were few events, which may have limited power.

The analysis of the rate of exacerbations by treatment and fibrinogen quartiles showed that rate ratio point estimates were generally in favor of FF/UMEC/VI versus both dual therapies in all quartile subgroups, with the most pronounced effect seen in the second quartile (fibrinogen ≥ 2.780 to < 3.280 g/L), and smaller between-treatment differences generally seen in the two highest quartiles. The analysis of exacerbation rates by treatment and fibrinogen threshold of 3.5 g/L showed a significant benefit of FF/UMEC/VI over FF/VI and UMEC/VI only in patients with fibrinogen < 3.5 g/L. The explanation for this is unclear. Due to the lack of data before Week 16, these results should be interpreted with caution as this limits inferences that may be made around treatment effect; there was a lower rate of treatment discontinuation in patients treated with triple versus dual therapy [3], possibly confounding any potential treatment effects. Finally, patients with higher fibrinogen levels had more exacerbations, and it is possible to speculate that these could be biologically different events that did not respond as well to ICS.

Fibrinogen is a known risk factor in CV disease, and fibrinogen levels have been shown to be higher in patients with CV disease than those without [18]. In our analyses, the highest fibrinogen quartile had similar incidence of CV events compared with the other quartiles, with only a small increase in annual event rate (0.16 event per patient-year in the highest quartile vs 0.13–0.15 in the other quartiles). Only SAEs and deaths were independently adjudicated in the IMPACT trial; therefore, the lack of an observable increase in AESIs with increasing levels of fibrinogen in our analysis could potentially be due to AEs being misclassified by the clinician as respiratory in nature when in fact they were CV events because of overlapping clinical symptoms between the diseases [19, 20].

Systemic inflammation has been associated with CV disease, cancer, and metabolic syndrome as well as COPD [9, 21–23]. The relationship between reduced fibrinogen and protection from exacerbations [9, 11, 24], suggests that there might be a low-grade inflammatory condition in the lung or extrapulmonary that predisposes patients to exacerbations. It is well known that ICS and LABA therapy can reduce exacerbations in patients with a low risk of exacerbations [25]. However, the absence of an effect of treatment on fibrinogen levels suggests that the site of this ongoing inflammatory response may not reside in the lung. It should be noted that patients with COPD may also suffer from comorbid diseases that could drive an increase in fibrinogen levels, such as cardiac failure or diabetes; therefore, higher fibrinogen levels may also be an indicator of a comorbidity [4].

The sample size for the IMPACT trial was large allowing for these subgroup analyses; however, there are limitations that should be considered when interpreting these data. Fibrinogen data were only collected at Week 16. This is supported by previous studies that had shown that treatment with oral steroids [16] or ICS/LABA [15] did not have an effect on fibrinogen levels. However, it is worth noting that the exacerbation incidence within the first 16 weeks was higher in patients who withdrew from the study compared with those who remained in the study. Furthermore, 2261 patients did not have fibrinogen data; had data been available for these patients, a different result may have been observed. Additionally, survival bias may mean that patients included in this analysis are not representative of a general patient population with COPD. Furthermore, overall data from the IMPACT trial indicates there was a clear separation between treatment arms for both moderate or severe exacerbations and mortality by Week 16, with ACM significantly lower for FF-containing therapies, which could have influenced the results [3].

## Conclusions

In this post hoc analysis of fibrinogen levels in the IMPACT trial, higher fibrinogen levels in patients with COPD were associated with a higher rate and risk of exacerbations. There appeared to be no association between fibrinogen levels and risk of death, although the lack of data prior to Week 16 and the relatively small number of deaths overall limit interpretation on risk of death, in addition to the assessment of any potential treatment effects. The incidence of AESIs was similar between fibrinogen levels, with pneumonia and CV events slightly more common at the highest fibrinogen quartile. Overall, our findings confirm the utility of fibrinogen as a predictive biomarker of exacerbation risk but not for risk of mortality.

## Supplementary Information

**Additional file 1**: ** Table 1.** Baseline characteristics and demographics by treatment and Week 16 fibrinogen quartile. **Table 2.** Baseline characteristics and demographics by treatment and Week 16 fibrinogen 3.5 g/L threshold. **Table 3.** COPD exacerbation history by treatment group and by withdrawal status at Week 16 (ITT population). **Table 4.** Analysis of fibrinogen levels at Week 16 by treatment group. **Table 5.** Incidence and rates of AESIs by fibrinogen quartile. **Figure 1.** Rate of on-treatment moderate/severe exacerbations from Week 16 by continuous fibrinogen level at Week 16; (A) moderate exacerbations; (B) moderate/severe exacerbations; (C) severe exacerbations. **Figure 2.** Time-to-first on-treatment COPD exacerbation from Week 16 by Week 16 fibrinogen 3.5 g/L threshold: (A) moderate exacerbations; (B) moderate/severe exacerbations; (C) severe exacerbations.

## Data Availability

Anonymized individual participant data and study documents can be requested for further research from www.clinicalstudydatarequest.com.

## Abbreviations

ACM: All-cause mortality
AE: Adverse event
AESI: Adverse events of special interest
ANOVA: Analysis of variance
BMD: Bone mineral density
COPD: Chronic obstructive pulmonary disease
CV: Cardiovascular
FEV_1_: Forced expiratory volume in 1 second
FDA: US Food and Drug Administration
FF: Fluticasone furoate
ICS: Inhaled corticosteroid
IOR: Interquartile range
IMPACT: InforMing the Pathway of COPD Treatment
ITT: Intent-to-treat
LABA: Long-acting β_2_-agonist
LRTI: Lower respiratory tract infection
SD: Standard deviation
SMQ: Standardized Medical Dictionary for Regulatory Activities query
UMEC: Umeclidinium
VI: Vilanterol

## References

[1] Singh D, Agusti A, Anzueto A, Barnes PJ, Bourbeau J, Celli BR, Criner GJ, Frith P, Halpin DMG, Han M (2019). Global strategy for the diagnosis, management, and prevention of chronic obstructive lung disease: the GOLD science committee report 2019. Eur Respir J.

[2] Global Initiative for Chronic Obstructive Lung Disease: Global strategy for the diagnosis, management and prevention of chronic obstructive pulmonary disease. 2021.

[3] Lipson DA, Barnhart F, Brealey N, Brooks J, Criner GJ, Day NC, Dransfield MT, Halpin DMG, Han MK, Jones CE (2018). Once-daily single-inhaler triple versus dual therapy in patients with COPD. N Engl J Med.

[4] Duvoix A, Dickens J, Haq I, Mannino D, Miller B, Tal-Singer R, Lomas DA (2013). Blood fibrinogen as a biomarker of chronic obstructive pulmonary disease. Thorax.

[5] Vestbo J, Rennard S (2010). Chronic obstructive pulmonary disease biomarker(s) for disease activity needed–urgently. Am J Respir Crit Care Med.

[6] Singh D, Roche N, Halpin D, Agusti A, Wedzicha JA, Martinez FJ (2016). Current controversies in the pharmacological treatment of chronic obstructive pulmonary disease. Am J Respir Crit Care Med.

[7] Anzueto A (2010). Impact of exacerbations on COPD. Eur Respir Rev.

[8] Vestbo J, Edwards LD, Scanlon PD, Yates JC, Agusti A, Bakke P, Calverley PM, Celli B, Coxson HO, Crim C (2011). Changes in forced expiratory volume in 1 second over time in COPD. N Engl J Med.

[9] Miller BE, Tal-Singer R, Rennard SI, Furtwaengler A, Leidy N, Lowings M, Martin UJ, Martin TR, Merrill DD, Snyder J (2016). Plasma fibrinogen qualification as a drug development tool in chronic obstructive pulmonary disease. Perspective of the Chronic Obstructive Pulmonary Disease Biomarker Qualification Consortium. Am J Respir Crit Care Med.

[10] Stockley RA, Halpin DMG, Celli BR, Singh D (2019). Chronic obstructive pulmonary disease biomarkers and their interpretation. Am J Respir Crit Care Med.

[11] Mannino DM, Tal-Singer R, Lomas DA, Vestbo J, Graham Barr R, Tetzlaff K, Lowings M, Rennard SI, Snyder J, Goldman M (2015). Plasma fibrinogen as a biomarker for mortality and hospitalized exacerbations in people with COPD. Chronic Obstr Pulm Dis.

[12] Kim TH, Oh DK, Oh YM, Lee SW, Do Lee S, Lee JS (2018). Fibrinogen as a potential biomarker for clinical phenotype in patients with chronic obstructive pulmonary disease. J Thorac Dis.

[13] Dickens JA, Miller BE, Edwards LD, Silverman EK, Lomas DA, Tal-Singer R (2011). COPD association and repeatability of blood biomarkers in the ECLIPSE cohort. Respir Res.

[14] Pascoe SJ, Lipson DA, Locantore N, Barnacle H, Brealey N, Mohindra R, Dransfield MT, Pavord I, Barnes N (2016). A phase III randomised controlled trial of single-dose triple therapy in COPD: the IMPACT protocol. Eur Respir J.

[15] Lomas DA, Lipson DA, Miller BE, Willits L, Keene O, Barnacle H, Barnes NC, Tal-Singer R (2012). An oral inhibitor of p38 MAP kinase reduces plasma fibrinogen in patients with chronic obstructive pulmonary disease. J Clin Pharmacol.

[16] Lomas DA, Silverman EK, Edwards LD, Locantore NW, Miller BE, Horstman DH, Tal-Singer R (2009). Serum surfactant protein D is steroid sensitive and associated with exacerbations of COPD. Eur Respir J.

[17] Celli BR, Anderson JA, Brook R, Calverley P, Cowans NJ, Crim C, Dixon I, Kim V, Martinez FJ, Morris A (2019). Serum biomarkers and outcomes in patients with moderate COPD: a substudy of the randomised SUMMIT trial. BMJ Open Respir Res.

[18] Stec JJ, Silbershatz H, Tofler GH, Matheney TH, Sutherland P, Lipinska I, Massaro JM, Wilson PF, Muller JE, D'Agostino RB (2000). Association of fibrinogen with cardiovascular risk factors and cardiovascular disease in the Framingham Offspring Population. Circulation.

[19] McCullough PA, Hollander JE, Nowak RM, Storrow AB, Duc P, Omland T, McCord J, Herrmann HC, Steg PG, Westheim A (2003). Uncovering heart failure in patients with a history of pulmonary disease: rationale for the early use of B-type natriuretic peptide in the emergency department. Acad Emerg Med.

[20] Rutten FH, Cramer MJ, Lammers JW, Grobbee DE, Hoes AW (2006). Heart failure and chronic obstructive pulmonary disease: an ignored combination?. Eur J Heart Fail.

[21] Barnes PJ (2016). Inflammatory mechanisms in patients with chronic obstructive pulmonary disease. J Allergy Clin Immunol.

[22] Cirillo P, Sautin YY, Kanellis J, Kang DH, Gesualdo L, Nakagawa T, Johnson RJ (2009). Systemic inflammation, metabolic syndrome and progressive renal disease. Nephrol Dial Transplant.

[23] Van't Klooster CC, Ridker PM, Hjortnaes J, van der Graaf Y, Asselbergs FW, Westerink J, Aerts J, Visseren FLJ (2019). The relation between systemic inflammation and incident cancer in patients with stable cardiovascular disease: a cohort study. Eur Heart J.

[24] Ronnow SR, Sand JMB, Langholm LL, Manon-Jensen T, Karsdal MA, Tal-Singer R, Miller BE, Vestbo J, Leeming DJ (2019). Type IV collagen turnover is predictive of mortality in COPD: a comparison to fibrinogen in a prospective analysis of the ECLIPSE cohort. Respir Res.

[25] Martinez FJ, Vestbo J, Anderson JA, Brook RD, Celli BR, Cowans NJ, Crim C, Dransfield M, Kilbride S, Yates J (2017). Effect of fluticasone furoate and vilanterol on exacerbations of chronic obstructive pulmonary disease in patients with moderate airflow obstruction. Am J Respir Crit Care Med.

