# Supplementary material for: InforMing the PAthway of COPD Treatment (IMPACT) trial: fibrinogen levels predict risk of moderate or severe exacerbations
Source: Respir Res. 2021 Apr 28;22:130. doi: 10.1186/s12931-021-01706-y (PMC8080358; doi:10.1186/s12931-021-01706-y)
Supplement: Supplementary file 1 — Additional file 1: Table 1. Baseline characteristics and demographics by treatment and Week 16 fibrinogen quartile. Table 2. Baseline characteristics and demographics by treatment and Week 16 fibrinogen 3.5 g/L threshold. Table 3. COPD exacerbation history by treatment group and by withdrawal status at Week 16 (ITT population). Table 4. Analysis of fibrinogen levels at Week 16 by treatment group. Table 5. Incidence and rates of AESIs by fibrinogen quartile. Figure 1. Rate of on-treatment moderate/severe exacerbations from Week 16 by continuous fibrinogen level at Week 16; (A) moderate exacerbations; (B) moderate/severe exacerbations; (C) severe exacerbations. Figure 2. Time-to-first on-treatment COPD exacerbation from Week 16 by Week 16 fibrinogen 3.5 g/L threshold: (A) moderate exacerbations; (B) moderate/severe exacerbations; (C) severe exacerbations. [file 12931_2021_1706_MOESM1_ESM.docx]

**Additional materials**

**InforMing the PAthway of COPD Treatment (IMPACT) trial: Fibrinogen levels predict risk of moderate or severe exacerbations**

Dave Singh MD, Gerard J. Criner MD, Mark Dransfield MD, David M.G. Halpin MD, MeiLan K. Han MD, Peter Lange MD, Sally Lettis PhD, David A. Lipson MD, David Mannino MD, Neil Martin MD, Fernando J. Martinez MD, Bruce E. Miller PhD, Robert Wise MD, Chang-Qing Zhu PhD, David Lomas MD PhD

**Table 1. Baseline characteristics and demographics by treatment and Week 16 fibrinogen quartile**

|  | Quartile 1  n=2017  <2.780^a^ | | | Quartile 2  n=2025  ≥2.780 –<3.280^a^ | | | Quartile 3  n=2002  ≥3.280 –<3.830^a^ | | | Quartile 4  n=2050  ≥3.830^a^ | | |
| --- | --- | --- | --- | --- | --- | --- | --- | --- | --- | --- | --- | --- |
| Treatment | FF/UMEC/VI | FF/VI | UMEC/VI | FF/UMEC/VI | FF/VI | UMEC/VI | FF/UMEC/VI | FF/VI | UMEC/VI | FF/UMEC/VI | FF/VI | UMEC/VI |
| N | 828 | 770 | 419 | 807 | 820 | 398 | 855 | 779 | 368 | 872 | 804 | 374 |
| Age, years, mean (SD) | 65.6 (8.4) | 64.7 (8.4) | 65.3 (8.5) | 65.0 (8.3) | 65.3 (8.2) | 64.5 (8.2) | 65.3 (8.1) | 65.2 (8.2) | 65.1 (8.2) | 65.8 (8.2) | 65.4 (8.1) | 65.2 (8.2) |
| Male, n (%) | 618 (75) | 583 (76) | 315 (75) | 522 (65) | 560 (68) | 263 (66) | 534 (62) | 494 (63) | 241 (65) | 593 (68) | 530 (66) | 233 (62) |
| BMI, kg/m^2^, mean (SD) | 25.4 (5.6) | 25.7 (5.3) | 25.1 (5.0) | 26.2 (5.9) | 26.4 (5.6) | 26.5 (5.9) | 27.4 (6.1) | 27.0 (6.2) | 26.9 (6.2) | 27.5 (6.8) | 27.7 (6.6) | 28.0 (6.4) |
| Smoking status, n (%) |  |  |  |  |  |  |  |  |  |  |  |  |
| Current | 219 (26) | 214 (28) | 121 (29) | 265 (33) | 278 (34) | 133 (33) | 341 (40) | 302 (39) | 144 (39) | 330 (38) | 299 (37) | 156 (42) |
| Former | 609 (74) | 556 (72) | 298 (71) | 542 (67) | 542 (66) | 265 (67) | 514 (60) | 477 (61) | 224 (61) | 542 (62) | 505 (63) | 218 (58) |
| Lung function (post-bronchodilator) |  |  |  |  |  |  |  |  |  |  |  |  |
| FEV_1_ (L), mean (SD) | 1.336 (0.4948) | 1.365 (0.4865) | 1.342 (0.5012) | 1.296 (0.4855) | 1.327 (0.4913) | 1.327 (0.5080) | 1.284 (0.4957) | 1.280 (0.4810) | 1.244 (0.4584) | 1.241 (0.4698) | 1.242 (0.4704) | 1.236 (0.4444) |
| FEV_1_ % predicted, mean (SD) | 47.9 (14.9) | 48.3 (14.9) | 47.7 (15.1) | 46.5 (14.8) | 47.3 (14.7) | 46.4 (13.9) | 45.9 (15.0) | 46.0 (14.7) | 44.8 (14.6) | 44.4 (14.8) | 44.3 (14.0) | 44.7 (14.1) |
| FEV_1_/FVC ratio, mean (SD) | 0.474 (0.1176) | 0.472 (0.1169) | 0.468 (0.1235) | 0.469 (0.1169) | 0.477 (0.1177) | 0.484 (0.1239) | 0.473 (0.1179) | 0.476 (0.1187) | 0.474 (0.1241) | 0.471 (0.1191) | 0.473 (0.1192) | 0.478 (0.1195) |
| COPD exacerbations in previous year, n (%) |  |  |  |  |  |  |  |  |  |  |  |  |
| Moderate |  |  |  |  |  |  |  |  |  |  |  |  |
| 0 | 159 (19) | 122 (16) | 86 (21) | 141 (17) | 162 (20) | 67 (17) | 144 (17) | 145 (19) | 65 (18) | 166 (19) | 163 (20) | 68 (18) |
| 1 | 251 (30) | 252 (33) | 133 (32) | 281 (35) | 276 (34) | 144 (36) | 324 (38) | 257 (33) | 124 (34) | 287 (33) | 286 (36) | 123 (33) |
| ≥2 | 418 (50) | 396 (51) | 200 (48) | 385 (48) | 382 (47) | 187 (47) | 387 (45) | 377 (48) | 179 (49) | 419 (48) | 355 (44) | 183 (49) |
| Moderate/severe |  |  |  |  |  |  |  |  |  |  |  |  |
| 0 | 0 | 1 (<1) | 2 (<1) | 1 (<1) | 0 | 0 | 0 | 1 (<1) | 0 | 1 (<1) | 0 | 0 |
| 1 | 356 (43) | 333 (43) | 194 (46) | 351 (43) | 387 (46) | 180 (45) | 397 (46) | 350 (45) | 165 (45) | 388 (44) | 377 (47) | 160 (43) |
| ≥2 | 472 (57) | 436 (57) | 223 (53) | 455 (56) | 442 (54) | 218 (55) | 458 (54) | 428 (55) | 203 (55) | 483 (55) | 427 (53) | 214 (57) |
| Severe |  |  |  |  |  |  |  |  |  |  |  |  |
| 0 | 613 (74) | 603 (78) | 316 (75) | 600 (74) | 605 (74) | 302 (76) | 644 (75) | 580 (74) | 276 (75) | 639 (73) | 577 (72) | 285 (76) |
| 1 | 184 (22) | 148 (19) | 91 (22) | 177 (22) | 189 (23) | 85 (21) | 185 (22) | 174 (22) | 79 (21) | 205 (24) | 192 (24) | 70 (19) |
| ≥2 | 31 (4) | 19 (2) | 12 (3) | 30 (4) | 26 (3) | 11 (3) | 26 (3) | 25 (3) | 13 (4) | 28 (3) | 35 (4) | 19 (5) |
| Current medical conditions, n (%) |  |  |  |  |  |  |  |  |  |  |  |  |
| Angina pectoris | 25 (3) | 17 (2) | 8 (2) | 26 (3) | 35 (4) | 14 (4) | 32 (4) | 24 (3) | 17 (5) | 29 (3) | 39 (5) | 12 (3) |
| Myocardial infarction | 0 | 0 | 0 | 0 | 0 | 0 | 0 | 0 | 0 | 0 | 0 | 0 |
| Cardiovascular risk factors^b^, n (%) |  |  |  |  |  |  |  |  |  |  |  |  |
| Angina pectoris | 45 (5) | 42 (5) | 24 (6) | 62 (8) | 75 (9) | 23 (6) | 69 (8) | 51 (7) | 27 (7) | 59 (7) | 73 (9) | 30 (8) |
| Previous myocardial infarction | 41 (5) | 43 (6) | 28 (7) | 39 (5) | 53 (6) | 25 (6) | 69 (8) | 50 (6) | 19 (5) | 67 (8) | 68 (8) | 31 (8) |

Quartile 1, fibrinogen value <25th percentile; Quartile 2, fibrinogen value >25th percentile and <median; Quartile 3, fibrinogen value ≥median and <75th percentile; Quartile 4, fibrinogen value ≥75th percentile.

^a^fibrinogen level descriptor (g/L); ^b^risk factors includes past and current events.

BMI, body mass index; FF, fluticasone furoate; SD, standard deviation; UMEC, umeclidinium; VI, vilanterol.

**Table 2. Baseline characteristics and demographics by treatment and Week 16 fibrinogen 3.5 g/L threshold**

|  | Fibrinogen <3.5 g/L | | | Fibrinogen ≥3.5 g/L | | |
| --- | --- | --- | --- | --- | --- | --- |
| Treatment | FF/UMEC/VI | FF/VI | UMEC/VI | FF/UMEC/VI | FF/VI | UMEC/VI |
| N | 2079 | 1971 | 995 | 1283 | 1202 | 564 |
| Age, years, mean (SD) | 65.4 (8.3) | 65.2 (8.3) | 64.9 (8.3) | 65.4 (8.1) | 65.1 (8.1) | 65.2 (8.2) |
| Male, n (%) | 1415 (68) | 1386 (70) | 702 (71) | 852 (66) | 781 (65) | 350 (62) |
| BMI, kg/m^2^, mean (SD) | 26.1 (5.8) | 26.1 (5.5) | 26.0 (5.6) | 27.5 (6.7) | 27.6 (6.6) | 27.7 (6.4) |
| Smoking status, n (%) |  |  |  |  |  |  |
| Current | 656 (32) | 640 (32) | 318 (32) | 499 (39) | 453 (38) | 236 (42) |
| Former | 1423 (68) | 1331 (68) | 677 (68) | 784 (61) | 749 (62) | 328 (58) |
| Lung function (post-bronchodilator) |  |  |  |  |  |  |
| FEV_1_ (L), mean (SD) | 1.304 (0.4892) | 1.338 (0.4890) | 1.319 (0.4996) | 1.263 (0.4834) | 1.246 (0.4712) | 1.238 (0.4447) |
| FEV_1_ % predicted, mean (SD) | 46.9 (14.9) | 47.6 (14.8) | 46.6 (14.7) | 44.9 (14.8) | 44.5 (14.2) | 44.8 (14.1) |
| FEV_1_/FVC ratio, mean (SD) | 0.471 (0.1167) | 0.474 (0.1171) | 0.476 (0.1235) | 0.473 (0.1198) | 0.476 (0.1197) | 0.477 (0.1217) |
| COPD exacerbations in previous year, n (%) |  |  |  |  |  |  |
| Moderate |  |  |  |  |  |  |
| 0 | 379 (18) | 351 (18) | 186 (19) | 231 (18) | 241 (20) | 100 (18) |
| 1 | 703 (34) | 643 (33) | 335 (34) | 440 (34) | 428 (36) | 189 (34) |
| ≥2 | 997 (48) | 977 (50) | 474 (48) | 612 (48) | 533 (44) | 275 (49) |
| Moderate/severe |  |  |  |  |  |  |
| 0 | 1 (<1) | 2 (<1) | 2 (<1) | 1 (<1) | 0 | 0 |
| 1 | 914 (44) | 877 (44) | 456 (46) | 578 (45) | 561 (47) | 243 (43) |
| ≥2 | 1164 (56) | 1092 (55) | 537 (54) | 704 (55) | 641 (53) | 321 (57) |
| Severe |  |  |  |  |  |  |
| 0 | 1539 (74) | 1506 (76) | 751 (75) | 957 (75) | 859 (71) | 428 (76) |
| 1 | 462 (22) | 410 (21) | 216 (22) | 289 (23) | 293 (24) | 109 (19) |
| ≥2 | 78 (4) | 55 (3) | 28 (3) | 37 (3) | 50 (4) | 27 (5) |
| Current medical conditions, n (%) |  |  |  |  |  |  |
| Angina pectoris | 72 (3) | 62 (3) | 27 (3) | 40 (3) | 53 (4) | 24 (4) |
| Myocardial infarction | 0 | 0 | 0 | 0 | 0 | 0 |
| Cardiovascular risk factors^a^, n (%) |  |  |  |  |  |  |
| Angina pectoris | 151 (7) | 139 (7) | 56 (6) | 84 (7) | 102 (8) | 48 (9) |
| Previous myocardial infarction | 115 (6) | 131 (7) | 62 (6) | 101 (8) | 83 (7) | 41 (7) |

Quartile 1, fibrinogen value <25th percentile; Quartile 2, fibrinogen value >25th percentile and <median; Quartile 3, fibrinogen value ≥median and <75th percentile; Quartile 4, fibrinogen value ≥75th percentile.

^a^Risk factors includes past and current events.

BMI, body mass index; FF, fluticasone furoate; SD, standard deviation; UMEC, umeclidinium; VI, vilanterol.

**Table 3. COPD exacerbation history by treatment group and by withdrawal status at Week 16 (ITT population)**

|  | FF/UMEC/VI  N=4151 | FF/VI  N=4134 | UMEC/VI  N=2070 | Total  N=10,355 |
| --- | --- | --- | --- | --- |
| Withdrawn at Week 16 | | | | |
| Total number of moderate/severe exacerbations, n (%) |  |  |  |  |
| n | 341 | 547 | 311 | 1199 |
| 0 | 0 | 2 (<1) | 0 | 2(<1) |
| 1 | 165 (48) | 285 (52) | 149 (48) | 599 (50) |
| 2 | 125 (37) | 201 (37) | 106 (34) | 432 (36) |
| ≥3 | 51 (15) | 59 (11) | 56 (18) | 166 (14) |
| Remained in the study | | | | |
| Total number of moderate/severe exacerbations, n (%) |  |  |  |  |
| n | 3810 | 3587 | 1759 | 9156 |
| 0 | 2 (<1) | 3 (<1) | 2 (<1) | 7 (<1) |
| 1 | 1688 (44) | 1622 (45) | 782 (44) | 4092 (45) |
| 2 | 1704 (45) | 1567 (44) | 784 (45) | 4055 (44) |
| ≥3 | 416 (11) | 395 (11) | 191 (11) | 1002 (11) |

Number of COPD exacerbations reported in the 12 months prior to the screening visit.

COPD, chronic obstructive pulmonary disease; FF, fluticasone furoate; ITT, intent to treat; N, number of patients in treatment group; n, number of patients in subgroup; UMEC, umeclidinium; VI, vilanterol.

**Table 4. Analysis of fibrinogen levels at Week 16 by treatment group**

|  | FF/UMEC/VI  N=3362 | FF/VI  N=3173 | UMEC/VI  N=1559 |
| --- | --- | --- | --- |
| Mean (95% CI) g/L | 3.37 (3.33, 3.41) | 3.35 (3.31, 3.39) | 3.31 (3.25, 3.37) |
| FF/UMEC/VI vs dual therapy (as specified) |  |  |  |
| Difference (95% CI) | - | 0.02 (-0.03, 0.07) | 0.06 (0.00, 0.13) |
| Mean % difference | - | 1% | 2% |
| UMEC/VI vs FF/VI |  |  |  |
| Difference (95% CI) | - | -0.04 (-0.11, 0.02) | - |
| Difference as % of column mean | - | -1% | - |

CI, confidence interval; FF, fluticasone furoate; N, number of patients in treatment group; UMEC, umeclidinium; VI, vilanterol.

**Table 5. Incidence and rates of AESIs by fibrinogen quartile**

| AESI, n (%) | Week 16 fibrinogen | | | | | | | |
| --- | --- | --- | --- | --- | --- | --- | --- | --- |
|  | Quartile 1  N=2017 | | Quartile 2  N=2025 | | Quartile 3  N=2002 | | Quartile 4  N=2050 | |
|  | N (%) | Rate [#] | N (%) | Rate [#] | N (%) | Rate [#] | N (%) | Rate [#] |
| Total duration at risk from Week 16 (patient-years) | 1329.4 | | 1316.6 | | 1313.3 | | 1321.3 | |
| Anticholinergic syndrome (SMQ) | 49 (2) | 48.1 [64] | 38 (2) | 31.9 [42] | 49 (2) | 46.4 [61] | 50 (2) | 43.9 [58] |
| Asthma/bronchospasm (SMQ) | 11 (<1) | 8.3 [11] | 8 (<1) | 6.1 [8] | 8 (<1) | 6.1 [8] | 9 (<1) | 6.8 [9] |
| Cardiovascular effects | 147 (7) | 139.2 [185] | 143 (7) | 136 [179] | 136 (7) | 129.4 [170] | 168 (8) | 162.7 [215] |
| Decreased BMD and associated fractures | 26 (1) | 21.1 [28] | 26 (1) | 21.3 [28] | 29 (1) | 28.2 [37] | 33 (2) | 32.5 [43] |
| Effects on potassium | 11 (<1) | 8.3 [11] | 11 (<1) | 8.4 [11] | 11 (<1) | 8.4 [11] | 5 (<1) | 3.8 [5] |
| Gastrointestinal obstruction (SMQ) | 2 (<1) | 1.5 [2] | 4 (<1) | 3.0 [4] | 4 (<1) | 3.0 [4] | 2 (<1) | 1.5 [2] |
| Hyperglycemia/new onset DM (SMQ) | 54 (3) | 42.9 [57] | 49 (2) | 40.3 [53] | 51 (3) | 44.9 [59] | 66 (3) | 55.2 [73] |
| Hypersensitivity | 54 (3) | 47.4 [63] | 67 (3) | 57.0 [75] | 71 (4) | 60.9 [80] | 49 (2) | 40.1 [53] |
| LRTI excluding pneumonia | 77 (4) | 69.2 [92] | 63 (3) | 58.5 [77] | 85 (4) | 71.6 [94] | 74 (4) | 61.3 [81] |
| Local steroid effects | 88 (4) | 75.2 [100] | 76 (4) | 66.8 [88] | 113 (6) | 103.6 [136] | 118 (6) | 112.0 [148] |
| Ocular effects | 13 (<1) | 10.5 [14] | 12 (<1) | 10.6 [14] | 15 (<1) | 13.7 [18] | 21 (1) | 18.9 [25] |
| Pneumonia | 106 (5) | 89.5 [119] | 94 (5) | 78.2 [103] | 97 (5) | 80.0 [105] | 115 (6) | 94.6 [125] |
| Tremor | 1 (<1) | 0.8 [1] | 2 (<1) | 1.5 [2] | 0 |  | 0 |  |
| Urinary retention | 3 (<1) | 2.3 [3] | 4 (<1) | 3.0 [4] | 5 (<1) | 5.3 [7] | 4 (<1) | 3.0 [4] |

Rate is event rate per 1000 patient-years, calculated as the number of events x 1000, divided by the total duration at risk. Quartile 1, fibrinogen value <25th percentile; Quartile 2, fibrinogen value >25th percentile and <median; Quartile 3, fibrinogen value ≥median and <75th percentile; Quartile 4, fibrinogen value ≥75th percentile. #, number of events; AESI, adverse event of special interest; BMD, bone mineral density; DM, diabetes mellitus; LRTI, lower respiratory tract infection; n, number of patients in subgroup; SMQ, Standardized MeDRA Query.

**Figure 1. Rate of on-treatment moderate/severe exacerbations from Week 16 by continuous fibrinogen level at Week 16; (A) moderate exacerbations; (B) moderate/severe exacerbations; (C) severe exacerbations**


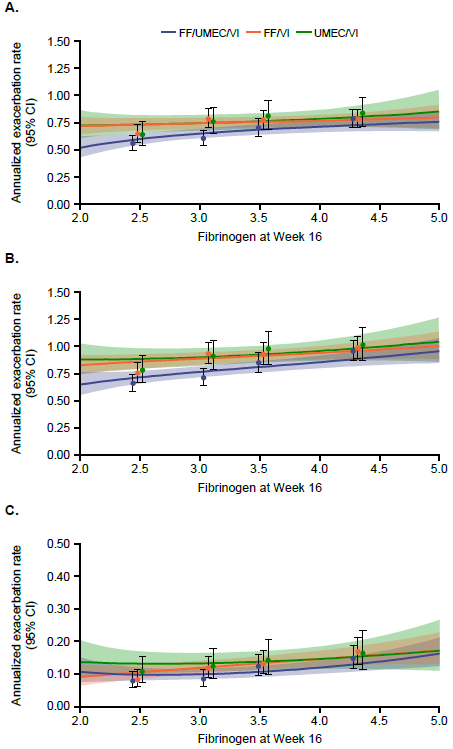


FF, fluticasone furoate, UMEC, umeclidinium; VI, vilanterol.

**Figure 2.** **Time-to-first on-treatment COPD exacerbation from Week 16 by Week 16 fibrinogen 3.5 g/L threshold: (A) moderate exacerbations; (B) moderate/severe exacerbations; (C) severe exacerbations**


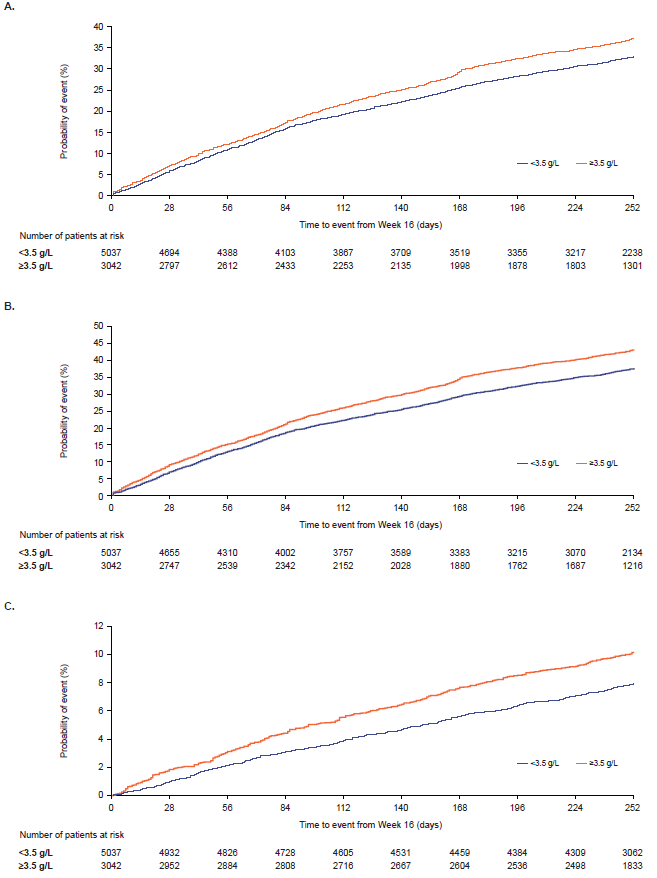


COPD, chronic obstructive pulmonary disease.
